# A novel small RNA regulates Locus of Enterocyte Effacement and site-specific colonization of enterohemorrhagic *Escherichia coli* O157:H7 in gut

**DOI:** 10.3389/fcimb.2024.1517328

**Published:** 2025-01-15

**Authors:** Runhua Han, Ye Qian, Chenguang Zheng

**Affiliations:** ^1^ Department of Chemistry, University of Manitoba, Winnipeg, MB, Canada; ^2^ School of Basic Medical Sciences, North China University of Science and Technology, Tangshan, China; ^3^ School of Public Health, North China University of Science and Technology, Tangshan, China

**Keywords:** enterohemorrhagic *Escherichia coli*, O157:H7, oxygen, sRNA (small RNA), gene regulation, LEE, site-specific colonization

## Abstract

Enterohemorrhagic *Escherichia coli* (EHEC) is a contagious foodborne pathogen that specifically colonizes the human large intestine, which is regulated by different environmental stimuli within the gut. Transcriptional regulation of EHEC virulence and infection has been extensively studied, while the posttranscriptional regulation of these processes by small RNAs (sRNAs) remains not fully understood. Here we present a virulence-regulating pathway in EHEC O157:H7, in which the sRNA EvrS binds to and destabilizes the mRNA of Z2269, a novel transcriptional regulator. In turn, Z2269 indirectly activates the expression of LEE (locus of enterocyte effacement) pathogenicity island through the master regulator Ler. Importantly, the expression of EvrS is modulated by environmental oxygen levels. EvrS also exhibits lower expression in the colon compared to the ileum, influencing the site-specific colonization of EHEC O157:H7 in mice. These results indicate that the oxygen status within the intestine may regulate the expression of EvrS, thereby modulating virulence factors of EHEC and contributing to successful infection *in vivo*. This study has broader implications for understanding sRNA functions in spatiotemporal virulence control of EHEC and may provide novel strategies to prevent EHEC infections.

## Introduction

*Escherichia coli* (*E. coli*) is the predominant facultative commensal of the human intestine (Leimbach et al., 2013). However, some *E. coli* strains have evolved pathogenic mechanisms through the acquisition of large clusters of virulence factors encoded on prophages and plasmids. These genetic elements enable *E. coli* to cause both intraintestinal and extraintestinal diseases, including meningitis, sepsis, diarrhea, and urinary tract infections (Kaper et al., 2004; Leimbach et al., 2013). Enterohemorrhagic *E. coli* (EHEC) is one of the most notorious pathogenic *E. coli*, responsible for a series of diseases ranging from mild diarrhea to life-threatening hemolytic uremic syndrome (Mellies et al., 2007; Wong et al., 2011). EHEC is typically ingested through contaminated food or water, where it survives the harsh conditions of the stomach and travels to the intestine. There, EHEC precisely adheres to the epithelial cells of the large intestine. Once attached, EHEC secretes a range of virulence factors that manipulate various intracellular pathways, enabling the pathogen to colonize, proliferate, and cause disease (Mellies et al., 2007; Wong et al., 2011).

Adherence of EHEC to intestinal epithelial cells is the first crucial step of intestinal colonization. This is accompanied by the development of a type of ultrastructural lesion called attaching and effacing (A/E) lesion, which causes the destruction of microvilli and the re-arrangement of cytoskeletal (Connolly et al., 2015). The formation of A/E lesions is largely dependent on a chromosomal pathogenicity island (PAI) termed the locus for enterocyte effacement (LEE) (Wong et al., 2011). LEE comprises 41 open reading frames organized in five major polycistronic operons (LEE1-5). LEE1, LEE2, and LEE3 harbor the genes encoding the major components of the type III secretion system (T3SS) that exports effector molecules. LEE1 also encodes Ler, a main transcriptional regulator of the LEE region that modulates the expression of other LEE operons. LEE4 encodes additional effector proteins translocated via T3SS to the host cell. The LEE5 operon encodes the Tir and intimin proteins, which are necessary for intimate attachment to the host epithelium (Mcdaniel et al., 1995). In addition to LEE, the virulence armamentarium of EHEC pathogenesis also involves Shiga toxin (Stx) and flagella (Wong et al., 2011). Stx is produced in the lower intestine, where it is translocated across the intestinal epithelium. Once in the bloodstream, it targets vascular endothelial cells, inducing necrosis or apoptosis by inhibiting protein synthesis (Pacheco and Sperandio, 2012). Stx has also been shown to promote EHEC adhesion to host epithelial cells by upregulating surface expression of phosphatidylethanolamine and nucleolin (Robinson et al., 2006). Flagella act as adhesive appendages during the initial phase of EHEC colonization, facilitating attachment and enhancing the bacterium’s motility within the intestine. This motility provides a crucial advantage by helping EHEC reach favorable niches while avoiding less hospitable areas (Mahajan et al., 2009; Chaban et al., 2015).

The production of virulence factors in pathogens may reduce bacterial growth rates under certain conditions and simultaneously trigger the host’s immune response (Sturm et al., 2011). Therefore, the timing of expression of virulence genes in EHEC must be tightly controlled. Indeed, EHEC expertly navigates complex environmental cues, such as fluctuations in oxygen, pH, temperature, and nutrient availability, to effectively colonize and infect their hosts (Connolly et al., 2015; Cordonnier et al., 2016; Lackraj et al., 2016; Le Bihan et al., 2017; Gelalcha et al., 2022). Extensive research has revealed a plethora of proteinaceous regulators that sense these signals in the infected gut to control transcription of the virulence genes, especially LEE (Connolly et al., 2015). Notably, posttranscriptional regulatory mechanisms that converge on the EHEC pathogenicity by small regulatory RNAs (sRNAs) is becoming increasingly recognized (Sauder and Kendall, 2018; Sy and Tree, 2021). sRNAs (sRNA) are heterogeneous molecules ranging from ∼50 to 500 nucleotides in length and recognized as important players in many physiological and adaptive responses (Jørgensen et al., 2020). Their synthesis is tightly regulated and often induced by a specific stress or virulence condition. Most sRNAs function as regulators by directly base pairing with *trans*-encoded mRNAs over short regions with imperfect complementarity (Felden and Augagneur, 2021). The canonical mechanism of sRNA-mediated regulation involves interaction with the 5’ untranslated region (5’UTR) or the region near the ribosome binding site (RBS) of the target mRNAs. In this process, the sRNA sequesters the RBS, thereby affecting translation and/or mRNA stability (Gottesman and Storz, 2011; Papenfort et al., 2013). A few recent studies also revealed that sRNAs can directly interact within the coding region and affect the stability of a target mRNA (Pfeiffer et al., 2009; Melson and Kendall, 2019; Villa et al., 2021). Transcriptome sequencing (RNA-seq) and UV-induced RNA-protein cross-linking and analysis of cDNA by high-throughput sequencing (CRAC) experiments revealed hundreds of sRNAs specific to EHEC, many of which are encoded within prophages (Tree et al., 2014; Gruber and Sperandio, 2015; Han et al., 2017). One of these sRNAs, StxS, modulates the expression of Shiga toxin 1 and the general stress response sigma factor RpoS through direct interaction of their mRNAs (Sy et al., 2020). Several other sRNAs (e.g., Esr055, Esr41, MavR, DicF) also play important roles in EHEC pathogenesis by regulating different genes and pathways (Sudo et al., 2014; Han et al., 2017; Melson and Kendall, 2019; Sauder and Kendall, 2021). However, the mRNA targets and regulation mechanisms for the majority of sRNAs in EHEC are still unknown.

Oxygen is one of the most important signals that plays strong roles in flexibly modulating bacterial infection in the human intestine (Wallace et al., 2016). During its passage through the gastrointestinal tract, EHEC encounters relatively aerobic conditions that may suppress the expression of the LEE pathogenicity island and other virulence factors. This adaptation helps EHEC avoid constructing its T3SS in unfavorable environments, such as the small intestine, thereby conserving energy and preventing unnecessary activation of its virulence machinery. As EHEC moves into the large intestine, it encounters a drastically reduced oxygen environment (Espey, 2013). This change is largely due to the metabolic activities of the dense commensal microbiota that consume available oxygen, creating an anaerobic niche (Kendall and Sperandio, 2016). This reduction in oxygen levels can trigger the expression of LEE and T3SS, facilitating adherence to intestinal epithelial cells and enhancing its pathogenicity. Interestingly, a multi-copied sRNA, DicF, regulates the EHEC virulence through base pairing with the 5’UTR of the *pchA* mRNA under microaerobic condition (Melson and Kendall, 2019). This finding suggests that the ability to rapidly integrate oxygen sensing via sRNA-based regulation may be a key strategy for EHEC to establish infection. However, additional sRNA-mediated mechanisms of oxygen sensing and virulence in EHEC still remains uncovered.

In this study, we studied one of the previously discovered sRNAs and renamed it EvrS (**E** HEC **v** irulence **r** egulatory **s** RNA). We demonstrated that EvrS significantly impacts the bacterial adherence ability of EHEC O157:H7 through regulating the mRNA of Z2269, a novel transcriptional regulator that indirectly activates the expression of the master LEE regulator, Ler. We also found that the expression of EvrS is downregulated under oxygen-limited conditions, and its altered expression affects the site-specific colonization of EHEC O157:H7 in mice. Our study therefore enhances the understanding of the virulence mechanisms employed by EHEC and how this bacterium responds to diverse environmental stresses and regulates pathogenicity through sRNA-mediated control.

## Materials and methods

### Strains, growth conditions and genetic manipulations

The EHEC O157:H7 EDL933 strain and derivatives thereof were cultured at 37°C with shaking in Luria-Bertani (LB) broth or Dulbecco’s Modified Eagle’s Medium (DMEM; Thermo Scientific) unless otherwise specified. When necessary, antibiotics were used at the following concentrations: ampicillin (Sigma-Aldrich), 100 μg ml^-1^; chloramphenicol (Sigma-Aldrich), 15 μg ml^-1^; kanamycin (Sigma-Aldrich), 50 μg ml^-1^, nalidixic acid (Sigma-Aldrich), 50 μg ml^-1^.

Mutant strains were generated by substitution of a specific sRNA or gene with a kanamycin (*kan*) or a chloramphenicol acetyltransferase (*cat*) gene cassette using an optimized λ Red recombineering technology, as described previously (Datsenko and Wanner, 2000). Briefly, the chloramphenicol or kanamycin cassettes were amplified from pKD3 or pKD4, respectively, using primers carrying 39 bp of homology to the regions flanking the sRNA/gene to be deleted. The resulting PCR products were electroporated into bacteria carrying the plasmid pKD46 for homologous recombination. The mutants were selected on LB plates with chloramphenicol or kanamycin and verified by PCR amplification and Sanger sequencing. The pCP20 plasmids was then transformed into the mutant strains to remove the antibiotic cassettes.

The EvrS complementation strain (pEvrS) was constructed by cloning the EvrS sequence and its promoter region (100 bp upstream the transcriptional start site) into the SphI/HindIII digested pACYC184 plasmid. The resulting plasmid was then electroporated into the Δ*evrS* strain, and colonies were screened on LB plates with chloramphenicol. To construct the plasmid expressing the EvrS promoter-*lux* fusion, a PCR fragment corresponding to the EvrS promoter sequence (100 bp upstream the transcriptional start site and 10 bp downstream) was ligated into XhoI/BamHI digested pMS402 plasmid (Fan et al., 2020), which carries a promoter-less *luxCDABE* reporter gene. The recombinant plasmid was then transformed into the EDL933 WT strain, and colonies were screened on LB plates with kanamycin. The strain for Z2269 protein purification was constructed by cloning the *z2269* gene into the NdeI/BamHI digested pET28a vector. The resulting plasmid was then electroporated into the *E. coli* BL21 strain. To construct the Z2269 overexpression plasmid, the coding sequence of Z2269 was amplified by PCR and cloned into the EcoRI/BamHI digested pTrc99a vector. The recombined plasmid was transformed into the WT and Δ*ler* strains, and colonies were screened on LB plates with amplicillin. To tag the Z2269 protein with 3×FLAG, the fragment corresponding to coding sequence of Z2269 (excluding the stop codon) and 3×FLAG was amplified and cloned into the pTrc99a vector between the EcoRI/BamHI cut site. The resulting plasmid was then transformed into the Δ*z2269* strain.

To test the regulation of EvrS on the *z2269* gene, the translational fusion containing the EvrS binding site on the *z2269* mRNA and the *lacZ* reporter gene under the control of the arabinose promoter were generated in a similar way as previously described (Gruber and Sperandio, 2015). Briefly, the sequence of -100 bp from the translation start site to +48 bp of the *z2269* gene and *lacZ* was synthesized by IDT and cloned into the pBAD24 vector between the NheI and HindIII restriction cut sites. The sRNA overexpression plasmids were constructed by cloning the EvrS sequence into the SacII/HindIII cut site on pBAD33. The plasmids with translational fusion, sRNA overexpression, or both were transformed into the WT EDL933 strain for further experiments. Site-directed mutagenesis was performed with the QuikChange site-directed mutagenesis kit (NEB) using these plasmids as the templates.

To test the expression of EvrS *in vivo*, the *gfp* coding sequence (synthesized by IDT) was used to replace the *luxCDABE* on the pMS402 plasmid by Gibson assembly. The recombinant plasmid containing P_EvrS_-*gfp* fusion was introduced into a EDL933 WT strain that constitutively expresses a *mCherry* gene under the promoter of the *rpoD* gene on the chromosome. The promoter of *rpoD* was selected because its activity remains unchanged throughout the murine gut (Armetta et al., 2021). The strain carrying the constitutively expressed *mCherry* gene was constructed using the λ Red recombineering technology by inserting the sequence of *rpoD* promoter and *mCherry* coding sequence as well as the sequence of a kanamycin cassette (synthesized by IDT) on chromosome between T_5004191_ and A_5004192_, where no sRNA or coding sequence was identified in previous studies (Tree et al., 2014; Gruber and Sperandio, 2015; Han et al., 2017; Waters et al., 2017). The pCP20 plasmid was finally transformed into the insertion strain to remove the antibiotic cassette.

All the bacterial strains and plasmids used are listed in **Supplementary Table S1** and **Supplementary Table S2**. All the oligonucleotides used in this study are listed in **Supplementary Table S3**.

### RNA isolation and handling

Bacterial samples were collected by centrifugation and mixed with 1/5 volume of the 95% ethanol, 5% phenol, snap-frozen in liquid nitrogen and thawed on ice. Total RNA was extracted using the TRIzol Reagent (Thermo scientific) according to the manufacturer’s protocol. RNA samples were further digested by DNase I (New England Biolabs) and purified using the RNA Clean & Concentrator™-5 (Zymo Research). RNA quantity was measured using the NanoDrop-2000 spectrophotometer, and the integrity of the RNA was checked by gel electrophoresis.

### Rapid amplification of cDNA ends

RACE assays were carried out using the FirstChoice^®^ RLM-RACE Kit (Thermo Scientific) according to the manufacturer’s protocol. For 5’-RACE, 5 μg of total RNA was treated by Calf Intestine Alkaline Phosphatase and Tobacco Acid Pyrophosphatase to remove free 5’-phosphates and cap structures. A 45 base RNA adapter oligonucleotide was then ligated to the treated RNA population using T4 RNA ligase. sRNA-specific antisense primer and M-MLV reverse transcriptase were used for the 5’ end amplification. For 3’-RACE, a poly(A) tail was added to 3’-end of the total RNA using the Poly(A) Polymerase Tailing Kit (Epicentre). First strand cDNA was generated from poly(A)-tailed RNA using the 3’ RACE Adapter and M-MLV Reverse Transcriptase. The cDNA was then served as the template for 3’-end amplification using other 3’ RACE Primers which are complimentary to the anchored adapter, and a sRNA-specific antisense primer. Nested PCRs were carried out with nested primers provided by the kit and sRNA-specific antisense primers if necessary. The PCR products were cloned into pGEM^®^-T Vector Systems (Promega), and the inserts were identified by Sanger sequencing. The sequences of the oligonucleotides used for RACE are listed in **Supplementary Table S3**.

### Protein purification

The BL21 (DE3) strain carrying pET28a-Z2269 was grown in LB until the OD600nm reached 0.4~0.6. The protein was induced by 0.5 mM isopropyl β-D-1-thiogalactopyranoside (IPTG) for overnight at 18°C. The Z2269 protein was purified using the Ni-NTA Agarose (Qiagen) as described before (Han et al., 2022). The absorbance at 280nm for the purified protein was measured using Nanodrop, and the protein concentration was calculated using the extinction coefficient of the Z2269 approximated with Expasy.

### Electrophoretic mobility shift assay

To explore if Z2269 can directly bind to the promoter of *ler*, electrophoretic mobility shift assays (EMSAs) were conducted using the purified Z2269 protein and PCR-amplified DNA fragments corresponding to the *ler* promoter region (150 bp upstream the transcriptional start site). 40 ng of the purified PCR fragment was incubated with different amounts of purified Z2269 in the EMSA binding buffer [20 mM Tris (pH 7.5), 50 mM KCl, 5 mM MgCl_2_, and 10% (w/v) glycerol] at 37°C for 30 min. The samples were then electrophoresed for approximately 1.5 h at 90 V and 4°C on a 10% native polyacrylamide gel. The gel was then stained with Ethidium Bromide (Sigma-Aldrich) for 30 min and visualized using a UV transilluminator.

### Northern blotting

Northern blot assays were performed as previously described (Villa et al., 2021). 10 µg of total RNA was separated on 10%/7 M urea polyacrylamide gels at 120 V for 5~8 h. The gel was briefly rinsed in 0.5 × TBE buffer and electroblotted onto the Brightstar Plus nylon membranes (Applied Biosystems) at 15V for overnight and cross-linked with 254 nm UV light for 2 min. Crosslinked membranes were prehybridized for 1 h at 50°C in the PerfectHyb Plus hybridization buffer (Sigma-Aldrich). A ssDNA oligonucleotide specific to EvrS was labelled by γ-^32^P (PerkinElmer) and incubated with membranes in the PerfectHyb Plus hybridization buffer overnight at 50°C. After washes with 5×SSC once at 68°C for 15 min and with 1×SSC twice at 68°C for 20 min, signals were determined using a Typhoon FLA 7000 phosphorimager (GE Healthcare). The size of sRNA was determined by comparing its corresponding band to the ΦX174 DNA/HinfI Marker (Promega). The sequence of the specific probe is listed in **Supplementary Table S3**.

### Mouse infection experiments

All animal experiments were performed according to the standards set forth in the Guide for the Care and Use of Laboratory Animals (National Research Council, 2011). The study was approved by the Institutional Animal Care and Use Committee of the North China University of Science and Technology Affiliated Hospital.

BALB/c mice (female, 6-8 weeks old) were purchased from Beijing Vital River Laboratory Animal Technology Co. Ltd (Beijing, China). Groups of 7 mice were maintained with sterilized food and water *ad libitum* for 7 days and deprived of food 14 h prior to infection. Mice were then administered orally by pipetting feeding of 10^9^ CFU of WT^Nal^, Δ*evrS* ^Nal^, and pEvrS strains growing in logarithmic phase (at an OD600nm of 0.6) suspended in 100 μl phosphate-buffered saline (pH 7.4). The sterilized diet and sterilized water were returned after the infection. Mice were euthanized by aether asphyxiation 6 h later. The ileum and colon were dissected and weighed after removing the luminal contents. The tissues were washed and homogenized in cold, sterile 1×PBS. The bacterial CFU was determined by plating of serially diluted homogenates on LB agar plates with appropriate antibiotics (50 μg ml^-1^ nalidixic acid for WT^Nal^ and Δ*evrS* ^Nal^; 15 μg ml^-1^ chloramphenicol for pEvrS).

### Cell infection assays

Cell infection assays using human Caco-2 epithelial cells (ATCC HTB-37) were performed as described previously (Liu et al., 2020). Caco-2 cells were purchased from the Shanghai Institute of Biochemistry and Cell Biology of the Chinese Academy of Sciences (Shanghai, China) and maintained in Dulbecco’s Modified-Eagle’s Medium (DMEM) supplemented with 10% fetal bovine serum and 1% penicillin/streptomycin/gentamicin antibiotic mix and grown at 37°C and 5% CO_2_. WT, Δ*evrS*, and pEvrS strains were inoculated in DMEM and grown to an OD600nm of 0.6 at 37°C. Monolayers of Caco-2 cells were infected with bacteria cultured in DMEM at an m.o.i. (multiplicity of infection) of 100:1. After 3 h of incubation at 37°C in 5% CO2, cells were washed three times in pre-warmed PBS to remove unbound bacteria and lysed in 1 ml of 0.1% sodium dodecyl sulfate. Lysates were collected, diluted, and plated onto LB agar plates to determine bacterial colony-forming units (CFU). Each experiment was carried out at least three times.

### Growth curve measurement

WT, Δ*evrS* and pEvrS strains were grown overnight in LB. The next morning, the strains were washed in DMEM media, and normalized to the OD600nm of 0.03 in fresh DMEM media. The strains were grown aerobically and shaking at 37°C. At the indicated time points, the OD600nm of each strain was monitored by the SCI-UV1100 Spectrophotometer (SCILOGEX, LLC). The average values and standard errors were calculated from the results of three experiments.

### Real-time quantitative PCR

RT-qPCR was performed using the Luna^®^ Universal One-Step RT-qPCR Kit (New England Biolabs) and the Applied Biosystems 7500 Real-Time PCR systems (Applied Biosystems). 50 ng RNA of each sample was first converted to cDNA at 55°C for 10 min. The cDNA was then denatured at 95°C for 1 min, followed by 45 cycles of 95°C (310 sec) and 60°C (30 sec). All data were normalized to levels of housekeeping gene 16S rRNA (*rrsH*), and the relative expression level was calculated as fold change using the 2^-ΔΔCt^ method (Schmittgen and Livak, 2008). Each qRT-PCR analysis was performed in triplicates.

To evaluate the expression of EvrS *in vivo*, 6-week-old female BALB/c mice (n = 7) were infected intragastrically with 10^9^ CFU of the EDL933^Nal^ strain expressing P_EvrS_-fused GFP on the plasmid and *mCherry* on the chromosome (cells growing in DMEM with an OD600nm of 0.6 were collected). At 6 h following infection, the ileum and colon were removed aseptically. RNA was extracted, and the rRNAs were depleted using the Ribo-Zero Gold rRNA Removal Kit (Epicentre). qRT-PCR analysis was then carried out to compare the *gfp* expression in the two organs using the 2^-ΔΔCt^ method. The expression of the *mCherry* gene was used for normalization.

All the primers used for qRT-PCR analysis are listed in **Supplementary Table S3**.

### Chromatin immunoprecipitation

The pTrc99a vector carrying 3×FLAG-tagged *z2269* were constructed and transformed into the Δ*z2269* mutation strain. The bacterial cultures were grown to an OD600nm of 0.6 at 37°C in DMEM and further incubated with 1 mM IPTG for 30 min at 37°C to induce the expression of Z2269-3×FLAG. For crosslinking, the cells were incubated with 1% formaldehyde for 25 min at room temperature. The cells were then washed with ice-cold 1×TBS and resuspended in 800 μL IP buffer [100 mM Tris-HCl (pH 7.5), 200 mM NaCl, 1 mM EDTA, 50 µg/mL RNase A, 1 mM PMSF]. The cells were then sonicated on ice with 15 cycles of 10 s on/off at 30% amplitude to generate DNA fragments of approximately 200-500 bp. After sonication, the samples were subjected to centrifugation at 13,000 g for 20 min at 4°C. The supernatant was split into two (400 μL each), with one aliquot incubated with 20 μL monoclonal ANTI-FLAG antibody (Sigma-Aldrich) at 4°C for 4 h as the ChIP sample, and the other without incubation with any antibody (as the mock sample). Both the ChIP and mock samples were then incubated with 50 µl protein A magnetic beads (Thermo Scientific) for 3 h at 4°C. The beads were washed with 1×PBS buffer and resuspended in 200 µL elution buffer [50 mM Tris-HCl (pH 8.0), 10 mM EDTA, 1% SDS]. The elution samples were incubated with 50 µg/mL RNaseA (Thermo Scientific) for 1 h at 37°C, and then with 100 µg/mL proteinase K (Thermo Scientific) for another 1 h at 55°C. DNA in the elution was further extracted using the QIAamp DNA Micro Kit (Qiagen). RT-qPCR was then carried out to detect if the *ler* promoter fragment is enriched in the ChIP sample relative to the mock sample. The experiments were performed in three biological replicates.

### Enzyme-linked immunosorbent assay

Shiga toxin production was quantified using the RIDASCREEN Shiga toxin enzyme-linked immunosorbent assay (R-Biopharm) according to the manufacturer’s instructions. After growing the WT, Δ*evrS*, and pEvrS strains to an OD600nm of 0.6 in DMEM, supernatants were collected from 1 ml culture and sterilized by filtration (pore size, 0.22 µm). 100 µl of supernatants were incubated with 100 μl biotin-conjugated antibody for 60 minutes in a 96-well plate at room temperature. The wells were washed for five times using 300 µl wash buffer and incubated with 100 µl poly-streptavidin peroxidase conjugate for 30 minutes at room temperature. After washing five times, the wells were filled with 100 µl substrate, and the plate was incubated for 15 minutes in darkness at room temperature (20-25°C) before the addition of 50 µl stop reagent. Absorbances were measured at 450 nm using a Spark multilabel plate reader (Tecan). Three biological replicates were performed for each assay.

### RNA decay assay

Overnight WT and Δ*evrS* strains were diluted 1:100 in fresh DMEM medium and cultured at 37°C. After OD600nm reached to 0.6, rifampin was added to the cultures at a final concentration of 250 µg/ml. At 2.5-, 5-, 10-, 20-, and 30-min posttreatment, 2 ml samples of each culture were collected and stored in liquid nitrogen immediately. Total RNA extraction and RT-qPCR were performed as described above for each sample. The half-life of *z2269* was then calculated from three biological replicates.

### Motility assays

1 μl of logarithmic-phase bacterial culture grown in DMEM media was spotted onto the center of 0.3% LB agar plates. Kanamycin and chloramphenicol were added to the plates when necessary. The plates were incubated at 30°C for 10 h before taking the images of the plates.

### β-galactosidase activity assay

Translational β-galactosidase assays were performed as previously (Gruber and Sperandio, 2015). Overnight cultures of the strains containing the reporter plasmids were diluted in fresh DMEM medium and grown at 37°C until the OD600nm reached 0.6. The cell culture was then incubated with 0.2% arabinose for 30 min. 500 μl of culture was taken for β-galactosidase activity measurement using the β-Gal Assay Kit (Thermo Scientific) and a Spark multilabel plate reader (Tecan). β-Galactosidase activity is defined as (μmol of ortho-nitrophenol [ONP] formed min^-1^) × 10^6^/[OD600nm] × ml of cell suspension. Each experiment was conducted in three replicates.

### Luciferase activity assay

To determine the effects of environmental signals on EvrS expression, we performed a Luciferase activity assay. Bacteria harboring the promoter-luciferase fusion plasmid were grown in M9 minimal media until the OD600nm reached 0.6. Cells were further cultured with gentle shaking for another 30 min at 37°C with different “signals”, including pH 5.5, pH 8.5, low oxygen (microaerobic) condition, 200 nM biotin, 0.2% bile, 50 μM epinephrine, 50 μM norepinephrine, 500 μM indole, 200 mM NaCl, 200 µM α,α’-dipyridyl, 150 μM malate, 0.4% glucose, 20 µM riboflavin, 100 µM fucose, 10 mM N-acetylneuraminic acid, 10 mM N-acetylglucosamin, 1 mM pyruvate, 2.5 mM succinate, 2.5 mM fumarate, 100 mM acetate, 100 mM propionate, 100 mM butyrate, 5 mM choline, 150 nM vitamin B12, and 30 mM ethanolamine together with 150 nM vitamin B12. For the microaerobic condition, bacteria grown statically in M9 minimal media at 37°C in the presence of 5% CO_2_ in a 15 mL falcon tube with cap tightly closed until the OD600nm reached 0.6. After incubation with these signals/under these conditions, 100 μl of the bacterial culture was then taken to measure the luminescence intensity at a wavelength of 560 nm using a Spark multilabel plate reader (Tecan). The luciferase activity was normalized by dividing the luminescence intensities by OD600nm values. The average and standard error were calculated from the results of three experiments.

### Statistical analysis

All the data were shown as mean ± standard deviations (SD). The statistical analysis was performed with the Graphpad Prism software using the Student’s *t*-test. For mouse infection experiments, the Mann-Whitney rank-sum test was performed. *P* ≤ 0.05, ≤ 0.005, or ≤ 0.0005 was considered to be statistically significant (*), highly significant (**), or extremely significant (***), respectively.

## Results

### The sRNA EvrS modulates EHEC adherence to epithelial cells

**Figure 1:**
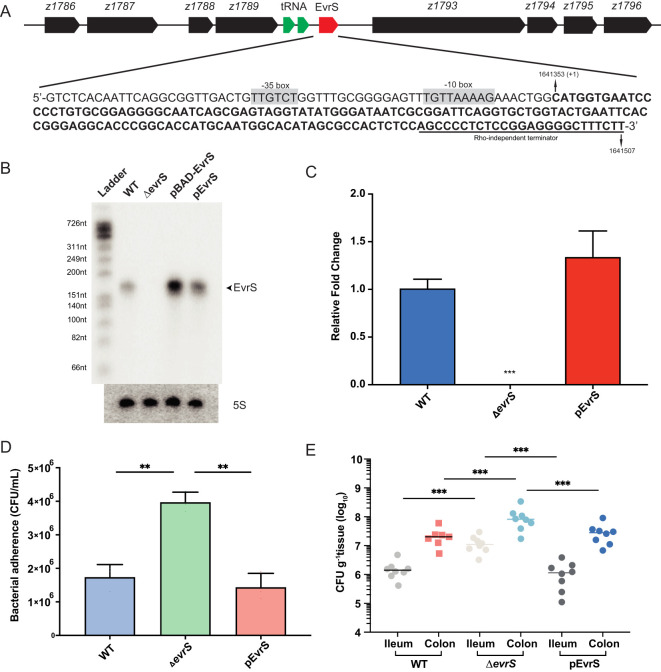
EvrS plays an essential role in pathogenesis of EHEC O157:H7. **(A)** Schematic diagram of the EvrS location on the chromosome of the EDL933 strain. The EvrS sequence is represented by bold. Putative -35 box and -10 box are highlighted in grey. The Rho-independent terminator is indicated by an underline. **(B)** Detection of EvrS by Northern blot analysis in the EDL933 wildtype (WT), EvrS deletion (Δ*evrS*), EvrS overexpression (pBAD-EvrS) (EvrS was induced with 0.2% arabinose for 10 min at OD600nm of 0.6), and EvrS complementation (pEvrS) strains at OD600nm of 0.6 grown in DMEM media. **(C)** RT-qPCR analysis of EvrS expression in EDL933 WT, EvrS deletion (Δ*evrS*), and EvrS complementation (pEvrS) strains. **(D)** Adherence capacity of the WT, Δ*evrS* and pEvrS strains to Caco-2 epithelial cells. CFU, colony-forming unit. **(E)** Adherence capacity of the WT, Δ*evrS*, and pEvrS strains in the ileum and colon of mice. CFU, colony-forming unit. For **(C, D)**, **, *P* ≤ 0.01; ***, *P* ≤ 0.001 (Student *t* test). For **(E)**, horizontal lines represent geometric means. ***, *P* ≤ 0.001 (Mann-Whitney rank-sum test).

Through a previous transcriptomic analysis, a small RNA (Esr023) was found in the prophage CP-933N on the EDL933 genome (Han et al., 2017). We renamed it EvrS (EHEC virulence regulatory sRNA) and determined its 5’- and 3’-ends on the chromosome of the EHEC O157:H7 EDL933 strain by RACE. The transcription start site of EvrS mapped to C_1641353_, and a putative Rho independent terminator was found upstream of T_1641507_ (**Figure 1A**). The length of EvrS was determined to be 154 bp, which is consistent with the size of the RNA band shown by Northern blot analysis (**Figure 1B**). The gene flanking EvrS on one side encodes a transcriptional regulator (*z1789*) and the other encodes a novel esterase (*z1793*) (**Figure 1A**). To test the function of EvrS, a mutant strain (Δ*evrS*) was constructed by homologous recombination in the EDL933 wild-type strain (WT), and complementation of EvrS was achieved using the pACYC184 plasmid expressing the sRNA (pEvrS). Northern blotting and qRT-PCR confirmed the absence of EvrS expression in the Δ*evrS* strain and the restored EvrS level in the pEvrS strain (**Figures 1C, D**).

The pathogenesis of EHEC O157:H7, to a considerable degree, depends on the adherence ability to intestinal epithelial cells (Wong et al., 2011). Therefore, we compared the adherence of the WT, Δ*evrS*, and pEvrS strains by incubating DMEM-grown bacteria with Caco-2 epithelial cells to analyze whether EvrS can affect bacterial adherence ability. We found that the adherence capacity of the Δ*evrS* strain to Caco-2 cells was significantly increased (by 5.25-fold) compared with the WT strain, which was restored in the pEvrS strain (**Figure 1E**). Importantly, no significant differences in growth rate were observed between the WT, Δ*evrS*, and pEvrS strains in DMEM (**Supplementary Figure S1A**). The deletion of EvrS did not alter significantly the expression of the surrounding ORFs as measured by qRT-PCR (**Supplementary Figure S1B**), indicating that the changed adherence ability of EvrS derivatives was not due to a growth difference or the altered expression of neighborhood genes.

We further tested the *in vivo* adherence capabilities of EvrS derivatives using a mouse model as described before (Liu et al., 2022). Groups of 7 BALB/c mice were infected with 10^9^ CFU bacteria (the WT, Δ*evrS*, and pEvrS strains) by oral gavage, and the number of bacteria recovered from the ileum and colon after 6 h was determined to evaluate bacterial colonization capacity. We found that the number of adhered bacteria to ileum and colon from Δ*evrS*-infected mice (1.06 × 10^7^ CFU g^-1^ tissue in ileum and 8.87 × 10^7^ CFU g^-1^ tissue in colon) was significantly higher than the adhered bacteria from WT-infected mice (1.78 × 10^6^ CFU g^-1^ tissue in ileum and 2.39 × 10^7^ CFU g^-1^ tissue in colon) (**Figure 1E**). Importantly, the number of bacteria that adhered to the colon from WT-infected mice was 13.40-fold higher than the bacteria adhered to the ileum (*P* < 0.01) (**Figure 1E**), which is consistent with the fact that EHEC preferentially colonizes in the large intestine. Moreover, the numbers of adhered bacteria from pEvrS-infected mice (2.25 × 10^7^ CFU g^-1^ tissue in the colon, and 1.36 × 10^6^ CFU g^-1^ tissue in the ileum) restored to the WT level (**Figure 1E**). Taken together, we conclude that EvrS is a negative regulator in EHEC O157:H7 pathogenesis by affecting the bacterial adherence ability.

### EvrS negatively regulates the expression of LEE via *ler*

**Figure 2:**
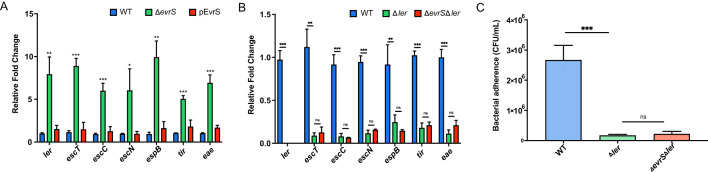
EvrS negatively affects locus of enterocyte effacement (LEE) gene expression through Ler. **(A)** RT-qPCR analysis of LEE expression in the WT, Δ*evrS*, and pEvrS strains. **(B)** RT-qPCR analysis of LEE expression in the WT, *ler* deletion (Δ*ler*) and EvrS+*ler* double deletion (Δ*evrS*Δ*ler*) strains. **(C)** Adherence capacity of WT, Δ*ler*, and Δ*evrS*Δ*ler* strains to Caco-2 epithelial cells. CFU, colony-forming unit. *, *P* ≤ 0.05; **, *P* ≤ 0.01; ***, *P* ≤ 0.001; ns, non-significant (Student *t* test).

The colonization ability of EHEC O157:H7 depends on a series of virulence factors (e.g., LEE), indicating that EvrS may affect the bacterial pathogenesis through regulating the expression of these factors (Foster, 2013; Carlson-Banning and Sperandio, 2018). To test this hypothesis, qRT-PCR was performed to measure the expression of seven representative LEE genes, including *ler* (the master regulator of LEE genes), *escT* (LEE1), *escC* (LEE2), *escN* (LEE3), *espB* (LEE 4), *eae* (intimin, LEE5), and *tir* (intimin receptor, LEE 5) in the WT, Δ*evrS* and pEvrS strains. As shown in **Figure 2A**, the expressions of all these LEE genes significantly increased in the absence of EvrS, whereas these changes were restored to wild-type levels in the pEvrS strain (**Figure 2A**). On the contrary, the bacterial motility and the production of Shiga toxin were not affected by the deletion and complementation of EvrS (**Supplementary Figures S2A**, **S2B**). These results suggested that the expression of EvrS only influences LEE expression but has no effect on Stx production and bacterial motility.

Considering that *ler* is one of the most important LEE regulators and that all LEE genes tested are affected by EvrS (**Figure 2A**), we assumed that EvrS may affect LEE expression via *ler*. To confirm this, we examined the EvrS regulation on LEE expression in the absence of *ler*. Deletion of *ler* in EHEC O157:H7 significantly reduced LEE gene expression and bacterial adherence to Caco-2 cells (**Figures 2A, B**), which is consistent with the role of Ler as a positive LEE regulator. Notably, the expression of LEE genes was comparable in the Δ*ler* strain and the Δ*evrS*Δ*ler* double deletion strain (**Figure 2A**). Furthermore, the cell adherence of the Δ*evrS*Δ*ler* strain was equivalent to the Δ*evrS* strain, which is much lower than the WT level (**Figure 2B**). These results suggested that the regulatory role of EvrS on bacterial adherence and LEE expression is mediated by Ler.

### EvrS regulates the expression of *z2269* mRNA by destabilizing the transcript

**Figure 3:**
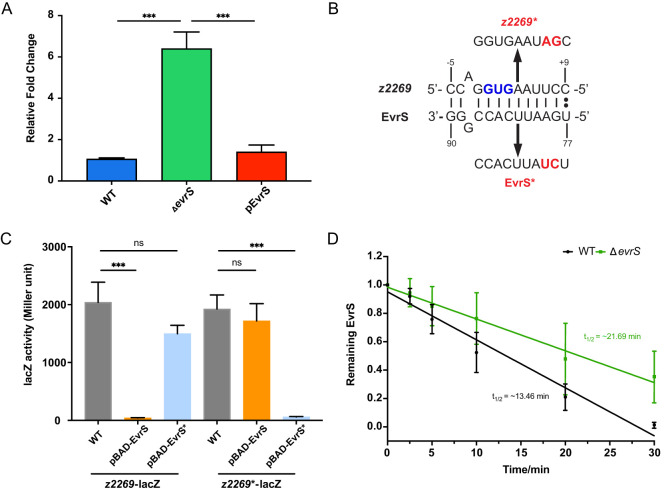
EvrS directly regulates and destabilizes the transcript of *z2269*. **(A)** RT-qPCR analysis of *z2269* expression in the WT, Δ*evrS*, and pEvrS strains. **(B)** Predicted binding sites between EvrS and *z2269* 5’UTR. The start codon (GUG) is indicated in blue. The mutations introduced on *z2269* or EvrS are indicated in red. **(C)** β-Galactosidase assays of the *z2269* translational reporter in the WT, pBAD-EvrS, or pBAD-EvrS* strains. **(D)** Half-life measurement of *z2269* in the WT and Δ*evrS* strains by RT-qPCR analysis. ***, *P* ≤ 0.001; ns, non-significant (Student *t* test).

To find out the direct target(s) of EvrS, we used Target RNA2 (Kery et al., 2014) and predicted 25 potential mRNA targets of the sRNA (**Supplementary Table S4**). To identify these targets experimentally, we selected thirteen candidates and carried out qRT-PCR to test the expression of these genes under different EvrS backgrounds. These candidates were selected based on their potential relevance to EHEC pathogenesis as reported in previous studies (they showed differential expression between infection and *in vitro* conditions) (Connolly et al., 2015; Yang et al., 2015; Woodward et al., 2019) and/or their likely regulation by EvrS [the predicted binding sites located in the translation initiation region (-20 to +20 relative to the start codon)]. One gene, *z2269*, showed increased expression in the Δ*evrS* strain compared to the WT strain, and the complementation of EvrS in the Δ*evrS* strain restored *z2269* expression to the wildtype level (**Figure 3A**). In contrast, the expressions of the other predicted EvrS target mRNAs did not change significantly upon EvrS deletion (**Supplementary Figure S3**). Interestingly, the predicted base pairing interactions between EvrS and *z2269* were around the translation start site of the mRNAs (**Figure 3B**), suggesting that EvrS may bind directly to the mRNA of *z2269* and affect its translation.

We further used a translational reporter system (Gruber and Sperandio, 2014) to test the *in vivo* regulatory outcomes of the EvrS-*z2269* interactions. In this assay, a DNA fragment spanning from -100 to +48 bp relative to the translation start site of the *z2269* mRNA was amplified and cloned upstream of the *lacZ* gene, under the control of the P_BAD_ promoter. This construct, along with another plasmid containing EvrS (also under the P_BAD_ promoter), was transformed into the wild-type strain. This setup allows the regulatory effect of EvrS on *z2269* translation to be assessed by measuring LacZ activity following induction of both the translational fusion and the sRNA (**Supplementary Figure S4**). Our results showed that the activity of *z2269-lacZ* fusion was repressed significantly upon EvrS overexpression (**Figure 3C**), indicating the translation of Z2269 is affected by EvrS. To examine whether the predicted binding sites are required for this regulation, we introduced mutations on the binding site without altering the codon preference (**Figure 3B**) and tested the effect of EvrS on the Z2269 fusion carrying the mutation (z2269*). We observed that the effect of EvrS on Z2269 was abolished when the mutation was introduced (**Figure 3C**). Moreover, the overexpression of EvrS* (carrying the corresponding mutations on the sRNA) significantly repressed the activity of z2269*-lacZ but has little effect on *z2269-lacZ*. These results indicate that the regulation of EvrS on *z2269* is mediated by the direct interaction between the two RNAs.

To reveal mechanistic insights into the regulation of EvrS regulation on *z2269*, an RNA decay assay was performed to determine whether EvrS affects the stability of *z2269* mRNA. The WT and Δ*evrS* cell cultures were treated with rifampicin to halt transcription. RNA samples were isolated at indicated time intervals posttreatment, and the abundance of *z2269* transcript was then determined by RT-qPCR analysis. As shown in **Figure 3D**, the half-life of the *z2269* mRNA was approximately 21.69 min in the Δ*evrS* strain, which is much longer than that in the WT strain (~13.46 min), indicating that the *z2269* transcript is more rapidly degraded in the presence of EvrS. Altogether, these results suggest that EvrS directly binds to *z2269* mRNA through the predicted binding sites and negatively regulates the stability of the transcript.

### Z2269 activates LEE expression and bacterial adherence through *ler*

**Figure 4:**
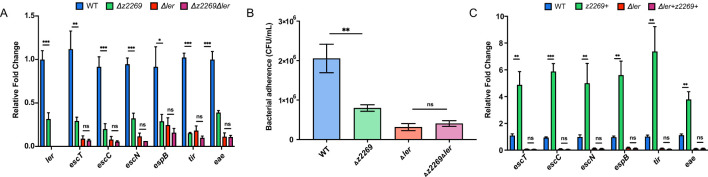
Z2269 activates locus of enterocyte effacement (LEE) gene expression and adherence to epithelial cells through Ler. **(A)** RT-qPCR analysis of LEE expression in WT, *z2269* deletion (Δ*z2269*), ler+z2269 double deletion (Δ*ler*Δz*2269*), and Δ*ler* strains. **(B)** Adherence capacity of WT, Δ*z2269*, Δ*ler*Δ*z2269* and Δ*ler* strains to Caco-2 epithelial cells. **(C)** RT-qPCR analysis of the expression of LEE expression in WT, WT strain overexpressing *z2269* (z*2269*+). *ler* deletion (Δ*ler*), and the Δ*ler* strain overexpressing *z2269* (Δ*ler*+z*2269*+). CFU, colony-forming unit. *, *P* ≤ 0.05; **, *P* ≤ 0.01; ***, *P* ≤ 0.001; ns, non-significant (Student *t* test).

Since *z2269* is the only identified target of EvrS, we assumed that the regulation of EvrS is mediated through this gene. We therefore investigated the contribution of *z2269* to the LEE expression and bacterial adherence. We found that the expression of LEE genes and bacterial adherence decreased significantly in the Δ*z2269* strain compared to WT by qRT-PCR and cell infection assay (**Figures 4A, B**). However, comparable LEE gene expression and bacterial adherence ability were observed in the Δ*ler* strain and Δ*z2269*Δ*ler* double deletion strain (**Figures 4A, B**). Moreover, overexpressing *z2269* only resulted in elevated expression of LEE genes in WT but not in the Δ*ler* strain (**Figure 4C**). These results implied that Z2269 activates EHEC adherence and LEE gene expression via Ler.

We next tested how *ler* is regulated by *z2269*. Through bioinformatic prediction, we found that Z2269 contains an N-terminal DNA-binding helix-turn-helix (HTH) motif and a C-terminal ligand-binding domain (**Supplementary Figure S5A**), implying that Z2269 may serve as a transcriptional regulator of *ler*. To test if Z2269 could regulate *ler* by directly interacting with its promoter, we performed an Electrophoretic Mobility Shift Assay (EMSA) by incubating the purified Z2269 protein (**Supplementary Figure S5B**) with the DNA fragment corresponding to the *ler* promoter. As shown in **Supplementary Figure S5C**, we didn’t observe the complex formation between Z2269 and the *ler* promoter fragment *in vitro*, suggesting that Z2269 is unable to bind *ler* promoter. However, this lack of binding could also be due to the absence of a required ligand in the EMSA reaction. To further understand this, a chromatin immunoprecipitation (ChIP)-qPCR assay was carried out to examine if Z2269 can bind *ler* promoter *in vivo*. Consistent with the EMSA result, we did not detect any significant enrichment of the *ler* promoter in the ChIP sample compared to the mock sample (**Supplementary Figure S5D**). These results suggest that Z2269 does not bind to the promoter region of *ler* but regulates the *ler* expression indirectly, probably through an unknown intermediary factor.

### The expression of EvrS is modulated by oxygen availability

**Figure 5:**
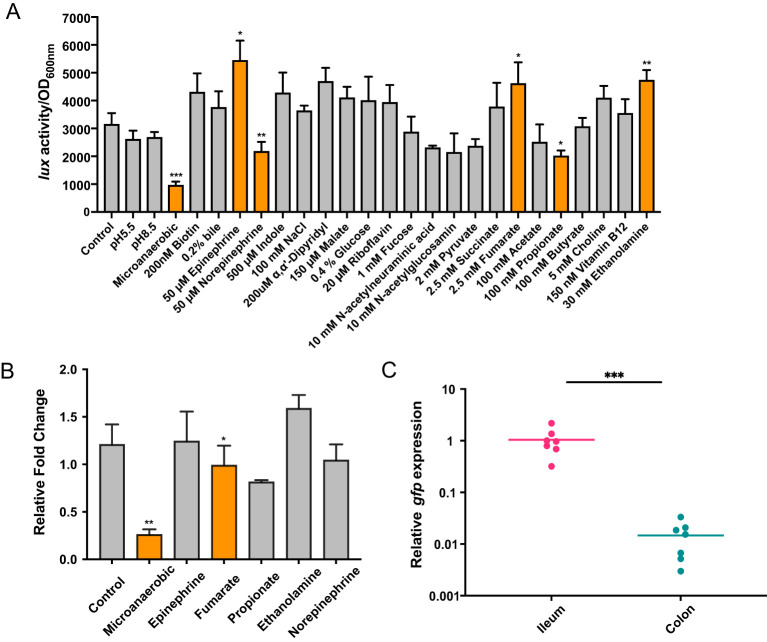
EvrS expression is affected by oxygen availability. **(A)** Activity of P_EvrS_-lux transcriptional fusions in EHEC EDL933 WT strain grown in M9 minimal media under the control condition (aerobic, pH 7.2) or under a series of other environmental conditions [pH 5.5, pH 8.5, low oxygen, or upon the incubation with 200 nM biotin, 0.2% bile, 50 μM epinephrine, 50 μM norepinephrine, 500 μM indole, 200 mM NaCl, 200 µM α,α’-dipyridyl, 150 μM malate, 0.4% glucose, 20 µM riboflavin, 100 µM fucose, 10 mM N-acetylneuraminic acid, 10 mM N-acetylglucosamin, 1 mM pyruvate, 2.5 mM succinate, 2.5 mM fumarate, 100 mM acetate, 100 mM propionate, 100 mM butyrate, 5 mM choline, 150 nM vitamin B12, or 30 mM ethanolamine (together with 150 nM vitamin B12)]. **(B)** RT-qPCR analysis of EvrS expression in WT under control or microaerobic conditions, or after adding 50 μM epinephrine/norepinephrine, 2.5 mM fumarate, 100 mM propionate, or 30 mM ethanolamine. **(C)** RT-qPCR analysis of *gfp* expression under EvrS promoter relative to *mCherry* under RpoD promoter in the ileum and colon of mice (n = 7). A higher *gfp*/*mCherry* value represents a higher EvrS expression. *, *P* ≤ 0.05; **, *P* ≤ 0.01; ***, *P* ≤ 0.001 (Student *t* test).

The rapid response of sRNA to environmental signals is important for EHEC adaption and colonization of different intestinal sites (González Plaza, 2020; Felden and Augagneur, 2021; Sy and Tree, 2021). To test which signal is involved in the regulation of EvrS, we amplified a DNA fragment containing the EvrS promoter region and fused with a promoter-less luciferase operon on the low-copy-number plasmid pMS402 (Fan et al., 2020). The fusion construct was introduced into the EHEC O157:H7 EDL933 strain and luminescence was measured during growth in M9 minimal medium by changing 26 previously reported “signals” that play significant roles in regulating virulence genes (especially LEE) in EHEC (Bansal et al., 2007; Kus et al., 2011; Kendall et al., 2012; Pacheco et al., 2012; Gonyar and Kendall, 2014; Yang et al., 2015; Carlson-Banning and Sperandio, 2016, 2018; Cordonnier et al., 2016; Lackraj et al., 2016; Le Bihan et al., 2017; Connors et al., 2018; Melson and Kendall, 2019; Liu et al., 2020, 2022). Compared to the control condition (aerobic, pH 7.2), the activity of the EvrS promoter significantly decreased by ~3.74 fold under microaerobic condition (**Figure 5A**). The EvrS promoter activity also significantly changed after incubating with 50 μM epinephrine/norepinephrine, 2.5 mM fumarate, 100 mM propionate, or 30 mM ethanolamine (**Figure 5A**), though the change was not so remarkable as that observed under the microaerobic condition. In contrast, no significant change was observed with other treatments (**Figure 5A**). To reassure this finding, we performed qRT-PCR analysis to compare the expression of EvrS under the control and microaerobic conditions, or upon the addition of epinephrine, fumarate, propionate, or ethanolamine. In consistent with the Luciferase activity assay results, our data showed that the EvrS level was significantly reduced by 3.85-fold under the microaerobic condition but didn’t show significant change in the presence of epinephrine/norepinephrine, fumarate, and ethanolamine (**Figure 5B**). The expression of EvrS also significantly changed after incubating with propionate, but it only showed a slightly reduced level (~ 0.81-fold) compared to the control. These results suggest that the expression of EvrS is mainly modulated by oxygen availability in the environment.

### EvrS contributes to the site-specific colonization of EHEC O157:H7 in mice large intestine

Considering EHEC normally encounters an oxygen gradient in the human intestinal tract, we proposed that the expression of EvrS might be differentially regulated in different intestinal niches with varying oxygen availability. The expression of EvrS during EHEC O157:H7 colonization in the intestine of mice was subsequently analyzed. To achieve this, RT-qPCR analysis was performed to evaluate the expression of EvrS in ileum and colon by measuring the *gfp* expression fused to the EvrS promoter. We found that the activity of EvrS promoter is significantly reduced in the colon compared with that in the ileum, as indicated by the reduced relative expression of *gfp* in the colon (*P* < 0.001) (**Figure 5C**). The differential expression of EvrS in mice intestine indicates that this sRNA may influence the site-specific colonization of EHEC O157:H7 in the large intestine. Indeed, in contrast to the WT strain, the difference between the adhered bacteria in the ileum and colon reduced to 8.27-fold in the Δ*evrS*-infected mice compared to the WT strain (the colonization of which is 13.4-fold higher in the colon than in the ileum), although the difference was still significant (*P* < 0.001) (**Figure 1E**). The adherence of the pEvrS strain in the colon was 15.17-fold higher than that in the ileum, which resembled the WT level (**Figure 1E**). Collectively, these results indicated that EvrS is differentially expressed between the ileum and colon, and contributes to the site-specific colonization of EHEC in the large intestine in mice.

## Discussion

The specific colonization of EHEC in the large intestine of mammals is highly regulated by different environmental stimuli in the intestine (Foster, 2013; Carlson-Banning and Sperandio, 2018). A deep understanding of this can provide insights into EHEC infection mechanisms and potential therapeutic interventions. Increasing evidence has indicated that these complex regulations possibly consist of multiple layers of regulation (transcriptional and posttranscriptional) coordinating responses to extracellular environments (Carlson-Banning and Sperandio, 2018; Sauder and Kendall, 2018; Melson and Kendall, 2019). These regulations are usually modulated by environmental changes in different niches. For instance, oxygen sensing is a key determinant for pathogens (including EHEC) in their ability to gauge their location within the host and effectively deploy their virulence arsenals (Wallace et al., 2016). During transit and colonization in the gastrointestinal (GI) tract, EHEC encounters an oxygen gradient with a decreasing trend from stomach to large intestine but faces an increasing oxygen trend from the anaerobic lumen to the epithelial border (Espey, 2013; Carlson-Banning and Sperandio, 2016). To date, several transcriptional regulators have been shown to play crucial roles in EHEC’s response to changes in oxygen levels in the environment. However, the mechanisms of oxygen sensing and virulence regulation by sRNAs in EHEC remain largely unknown.

**Figure 6:**
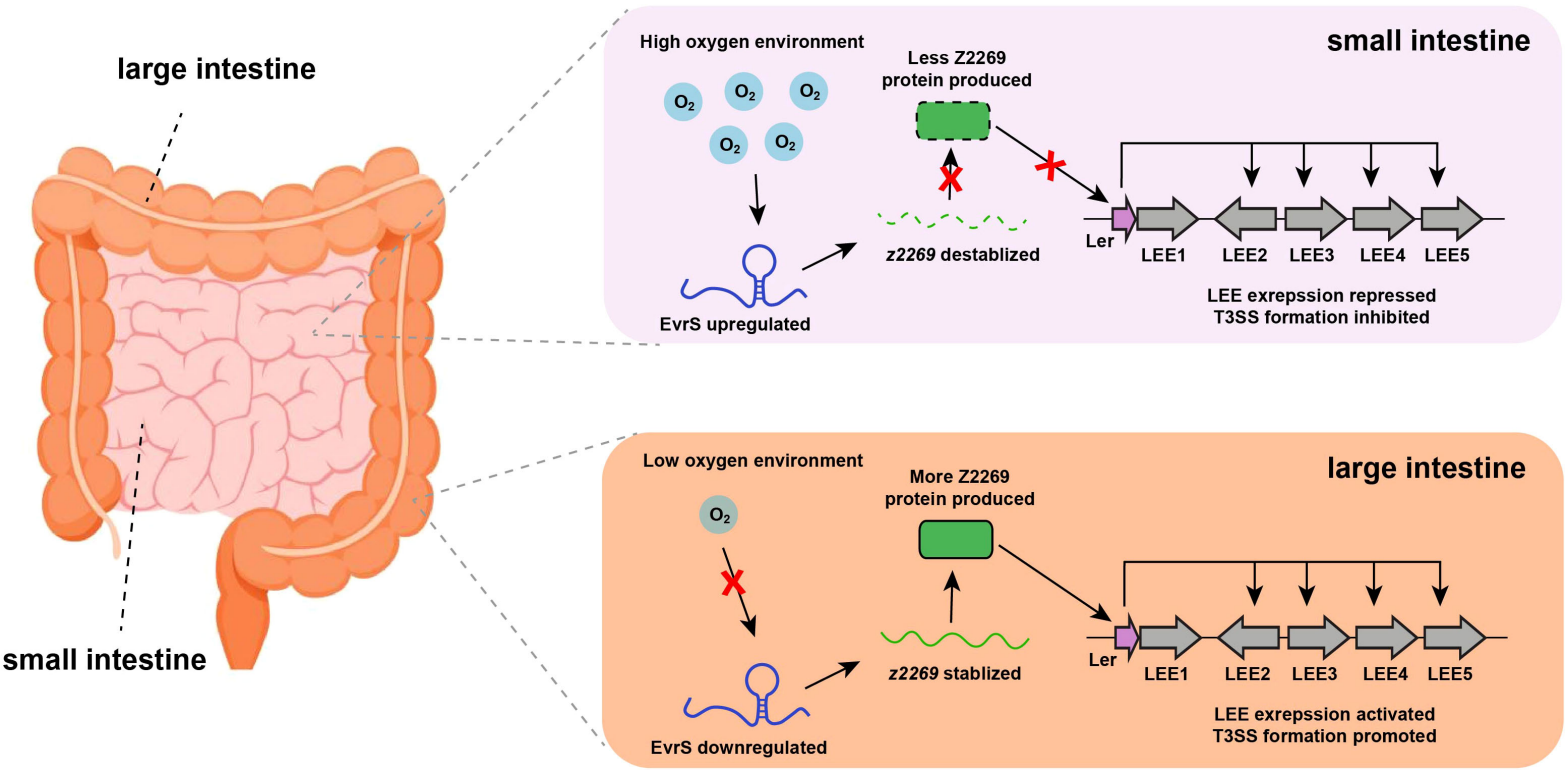
Proposed working model for EvrS regulation in different intestinal niches with varied oxygen availability.

Here, we demonstrated a signal transduction regulatory pathway in which EHEC utilizes a newly characterized sRNA to sense oxygen availability and regulate site-specific colonization in the host intestinal tract (**Figure 6**). Under oxygen-rich conditions, the expression of EvrS is upregulated, decreasing the abundance of *z2269* by destabilizing its transcript, leading to the further repression of LEE and prevention of bacterial adherence. This allows the bacteria to conserve energy and avoid unnecessary host immune responses at non-specific colonization sites (e.g., small intestine). When the bacteria enter an environment with lower oxygen levels (e.g., the large intestine), the expression of EvrS is downregulated, resulting in the upregulation of *z2269*. This, in turn, activates LEE expression and promotes bacterial adherence at specific colonization sites. Interestingly, the significant difference of colonized bacteria between ileum and colon was still observed in the Δ*evrS* strain, indicating this sRNA is not the only effector contributing to the preferential colonization of EHEC in the large intestine. Indeed, other factors (e.g., Esr055, BirA, Fur) have been revealed to promote the preferential colonization of EHEC in the colon in response to different stimuli (Yang et al., 2015; Han et al., 2017).

Notably, a recent study showed that another sRNA in EHEC, DicF, is also affected by oxygen availability *in vitro* and promotes LEE expression through the direct interaction with the *pchA*, a LEE modulator (Melson and Kendall, 2019). Although the mechanism of DicF was thoroughly investigated, it is still not fully understood how this sRNA responds to fluctuating oxygen levels in the intestine to affect the *in vivo* colonization. By characterizing EvrS, our study expands the knowledge of how sRNAs regulate the specific colonization of EHEC in the large intestine. Intriguingly, the expression of DicF was only observed under microaerobic conditions, whereas EvrS can be detected under both aerobic and microaerobic conditions (**Figures 1B**, **5**), suggesting other important roles of EvrS may exist under aerobic conditions. Unfortunately, we were unable to explain how oxygen modulates the expression of EvrS mechanistically. Upon examining the promoter sequence of the EvrS, we found a putative PdhR binding site and a putative CRP binding site (**Supplementary Figure S6A**). However, RT-qPCR analysis showed that both the mutant strains of *crp* and *pdhR* exhibited comparable EvrS levels to the WT strain (**Supplementary Figure S6B**). In addition, we examined the possible regulation of Fnr and ArcAB, two of the best-studied transcriptional regulators involved in adaptations to fluctuating oxygen levels in enteric pathogens (Salmon et al., 2003; Brown et al., 2022), on the expression of EvrS. As shown in **Supplementary Figure S6B**, no difference in EvrS level was observed in the Δ*fnr* and Δ*arcA* strains compared to the WT strain, suggesting that the regulation of EvrS by oxygen is through some unknown factors.

Compared to DicF, which has four copies on the chromosome of EHEC O157:H7, EvrS only has a single copy located in the prophage CP-933N. Prophages are widely distributed in different bacterial genomes, and the genes or sRNAs in prophages enable host bacteria to adapt to various external environments (Fröhlich and Papenfort, 2016; Altuvia et al., 2018; Pleška et al., 2018). Given the importance of phage-mediated horizontal gene transfer on the evolution of bacteria-phage interplay, we wondered if EvrS would be conserved in other strains. Bioinformatics analysis revealed that putative EvrS homologs were found in the published genomes of many other EHEC strains, exhibiting a high level of conservation (**Supplementary Figure S7**). Interestingly, the homolog of EvrS in the EHEC Sakai strain was found in the Hfq CRAC data, suggesting that the function of EvrS may depend on Hfq. However, whether and how Hfq (and other sRNA binding proteins) modulate EvrS regulation in EHEC needs further investigation. It is worth noting that EvrS was also found in some gastrointestinal and extraintestinal pathogenic bacteria such as Enteropathogenic *E. coli* (EPEC), Uropathogenic *E. coli* (UPEC), *Shigella flexneri*, and Neonatal meningitis-causing *E. coli* (NMEC). These results indicate that EvrS is a widespread regulatory element to modulate the virulence in a range of human pathogens, even though the regulatory roles of EvrS in these pathogens have not been revealed.

Finally, we were only able to confirm one RNA target that is directly regulated by base pairing with EvrS due to the limitations of experimental and computational techniques used in this study. Considering that sRNAs typically interact with and regulate the expression of multiple target genes (Jørgensen et al., 2020), an expanded targetome and regulatory role of EvrS is possible, which however needs further study in the near future.

## Data Availability

The original contributions presented in the study are publicly available. This data can be found here: https://doi.org/10.6084/m9.figshare.28067477.v1.
