# Correction: A novel small RNA regulates locus of enterocyte effacement and site-specific colonization of enterohemorrhagic *Escherichia coli* O157:H7 in gut

**DOI:** 10.3389/fcimb.2025.1642032

**Published:** 2025-07-10

**Authors:** Runhua Han, Ye Qian, Chenguang Zheng

**Affiliations:** ^1^ Department of Chemistry, University of Manitoba, Winnipeg, MB, Canada; ^2^ School of Basic Medical Sciences, North China University of Science and Technology, Tangshan, China; ^3^ School of Public Health, North China University of Science and Technology, Tangshan, China

**Keywords:** Enterohemorrhagic *Escherichia coli*, O157:H7, oxygen, sRNA (small RNA), gene regulation, LEE, site-specific colonization

Author Runhua Han was erroneously assigned to the affiliation “School of Basic Medical Sciences, North China University of Science and Technology, Tangshan, China”. This affiliation has now been removed for author Runhua Han. His sole affiliation is the “Department of Chemistry, University of Manitoba, Winnipeg, MB, Canada”.

Affiliation School of Public Health, North China University of Science and Technology, Tangshan, China was erroneously given as College of Public Health, North China University of Science and Technology, Tangshan, China.

The **Ethics statement** was erroneously given as “The animal study was approved by Institutional Animal Care and Use Committee of North China University of Science and Technology Affiliated Hospital. The study was conducted in accordance with the local legislation and institutional requirements”.

The correct **ethics statement** is “The animal experiments were approved by the Laboratory Animal Ethics Committee of North China University of Science and Technology and complied with the National Institute of Health’s Guide for the Care and Use of Laboratory Animals”.

The authors apologize for this error and state that this does not change the scientific conclusions of the article in any way. The original article has been updated.

