# Supplementary material for: A novel small RNA regulates Locus of Enterocyte Effacement and site-specific colonization of enterohemorrhagic Escherichia coli O157:H7 in gut
Source: Front Cell Infect Microbiol. 2025 Jan 15;14:1517328. doi: 10.3389/fcimb.2024.1517328 (PMC11774850; doi:10.3389/fcimb.2024.1517328)
Supplement: Supplementary file 1 [file DataSheet1.pdf]

Figure S1. Effect of EvrS on cell growth and expression of neighborhood genes in EHEC O157:H7. (**A**) The growth of the WT, Δ*evrS* and pEvrS strains aerobically in DMEM media. (**B**) RT-qPCR analysis of *z1789* and *z1793* expressions in the WT, Δ*evrS* and pEvrS strains. Data are presented as means ± SD of biological triplicates.

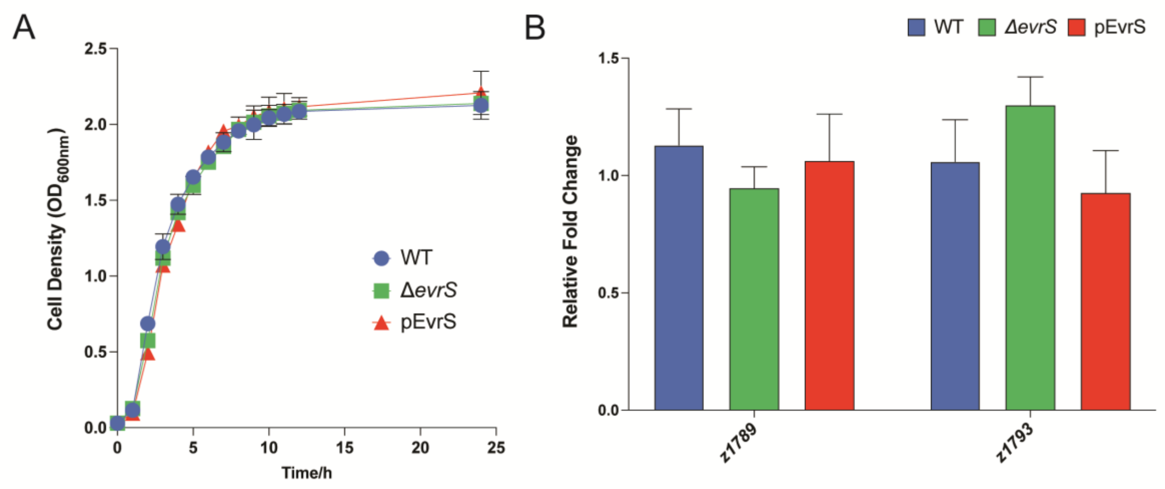

Figure S2. Effect of EvrS on mobility and Shiga toxin production in EHEC O157:H7. (**A**) Representative image of swimming motility of the WT, Δ*evrS* and pEvrS strains on motility agar. (**B**) Evaluation of Shiga toxin production in the WT, Δ*evrS* and pEvrS strains by ELISA assay. A higher absorbance at 450nm represents a higher Shiga toxin production.

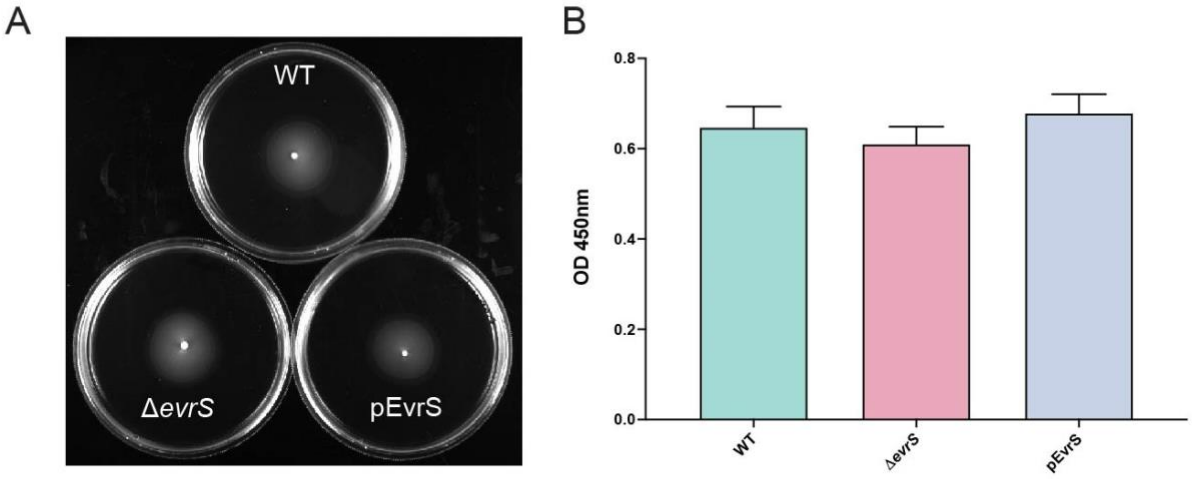

Figure S3. RT-qPCR analysis of selected potential targets of EvrS except for *z2269* in the WT and Δ*evrS* strains.

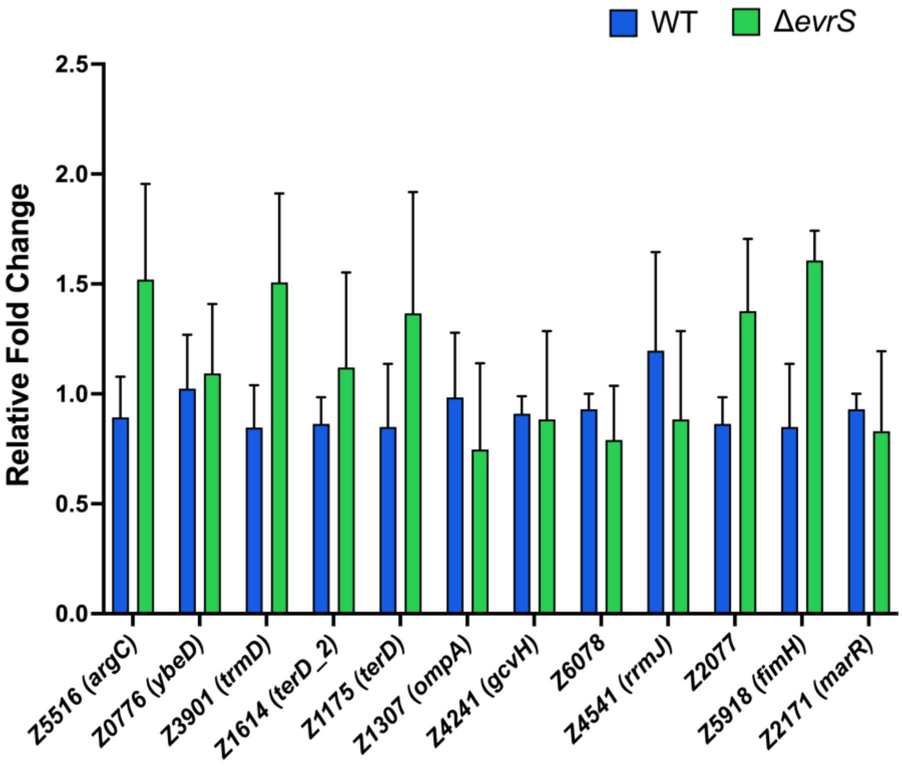

Figure S4. *In vivo* translational reporter system for detecting regulatory outcome of EvrS on *z2269* mRNA. 100 bp of the *z2269* 5' UTR region and 48 bp of the coding region was fused with *lacZ* gene on the pBAD24 vector, and the fusion is expressed under the arabinose inducible P_{BAD} promoter. The vector is transformed into the *E. coli* O157:H7 WT strain containing the pBAD33 vector, which expresses EvrS also under the P_{BAD} promoter. The regulatory outcome of EvrS on *z2269* mRNA is evaluated through measuring the LacZ activity after induction of both sRNA and the translational fusion.

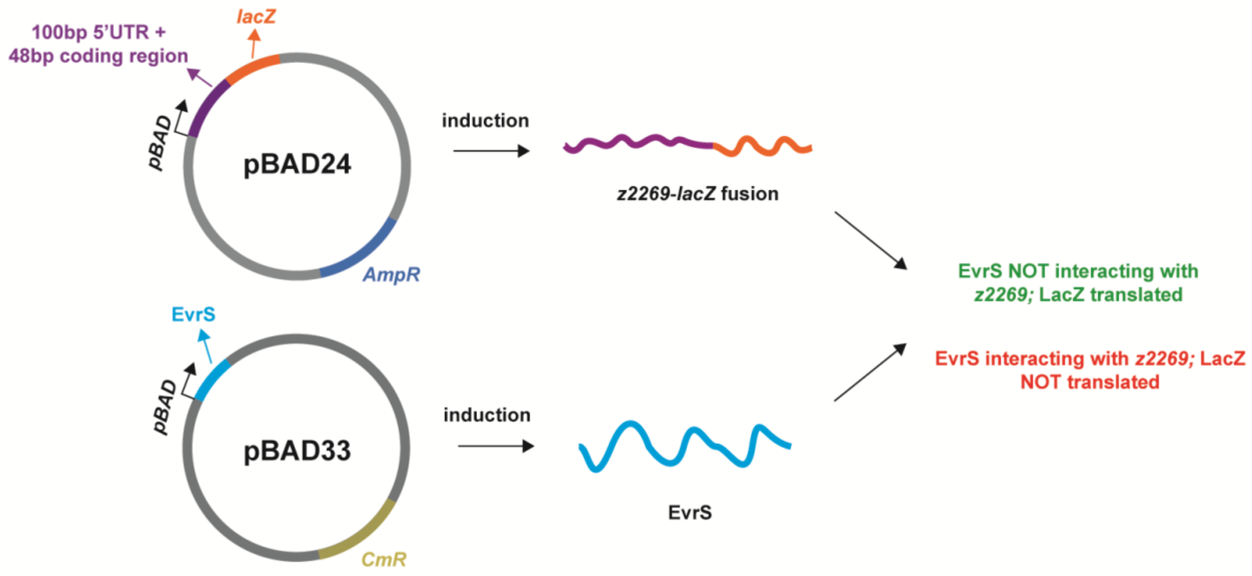

Figure S5. Z2269 is a novel LEE transcriptional regulator that does not directly bind to *ler* promoter. (**A**) Schematic showing the domain structure of the Z2269 protein. (**B**) Examination of purified Z2269 protein by SDS-PAGE followed by Coomassie staining. (**C**) No direct interaction between Z2269 protein and EvrS promoter region, as revealed by EMSA. EvrS promoter sequence were amplified by PCR, purified and added to the reaction mixtures at 40 ng for each reaction. Z2269 protein was added at final concentrations of 0.1 ~ 20 μM. No protein was added in the first lane as the control. (**D**) Enrichment of the *ler* promoter fragment in the Z2269-3×FLAG ChIP sample compared with the mock sample measured by RT-qPCR. Significance was determined using Student's *t*-test.

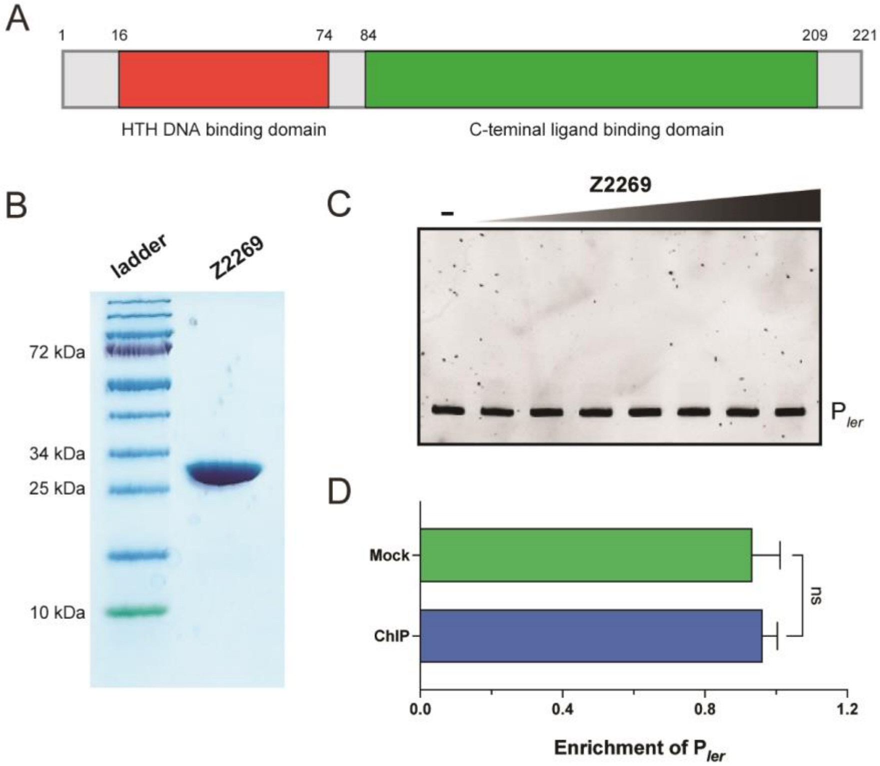

Figure S6. Examination of possible regulators of EvrS in EHEC O157:H7. (**A**) Schematic of putative Crp/PdhR binding sites on the promoter region of EvrS. (**B**) RT-qPCR analysis of EvrS expression in the WT, *crp* deletion (Δ*crp*), *pdhR* deletion (Δ*pdhR*), *fnr* deletion (Δ*fnr*), *arcA* deletion (Δ*arcA*) strains.

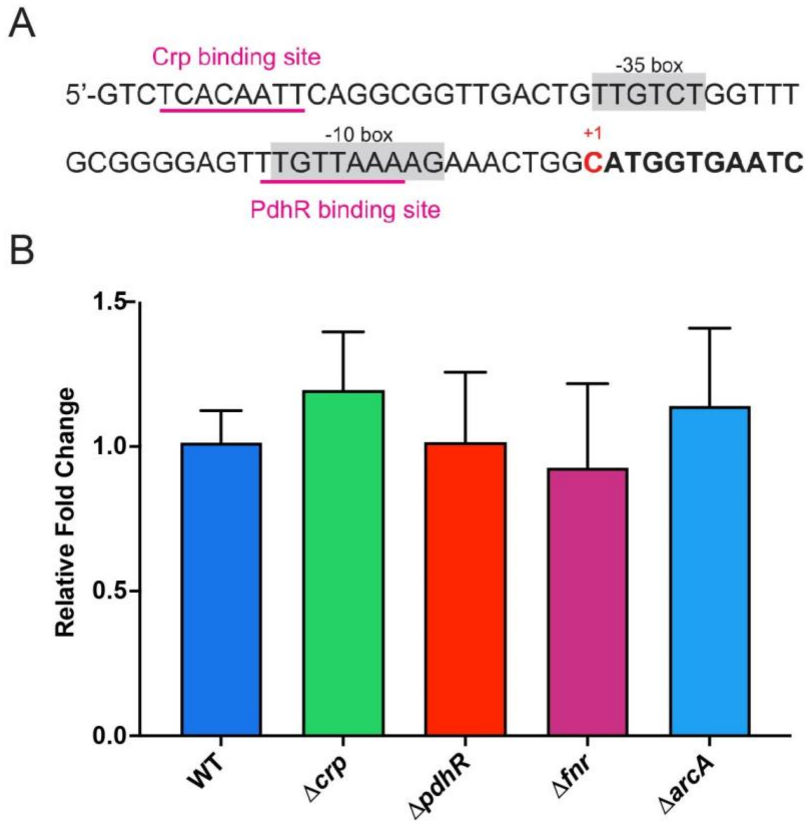

Figure S7. Alignment of the EvrS sequences. Sequences used for this alignment are from EHEC O157:H7 EDL933 strain, EHEC O157:H7 Sakai strain, EHEC O26:H11 11368 strain, EHEC O111:H- 11128 strain, EPEC O55:H7 CB9615 strain, EPEC O145:H28 RM11514 strain, EAEC O104:H4 2009EL_2071 strain, UPEC O6:K2:H1 CFT073 strain, NMEC O18:K1 RS218 strain, *Shigella flexneri* 2a 2457T strain.

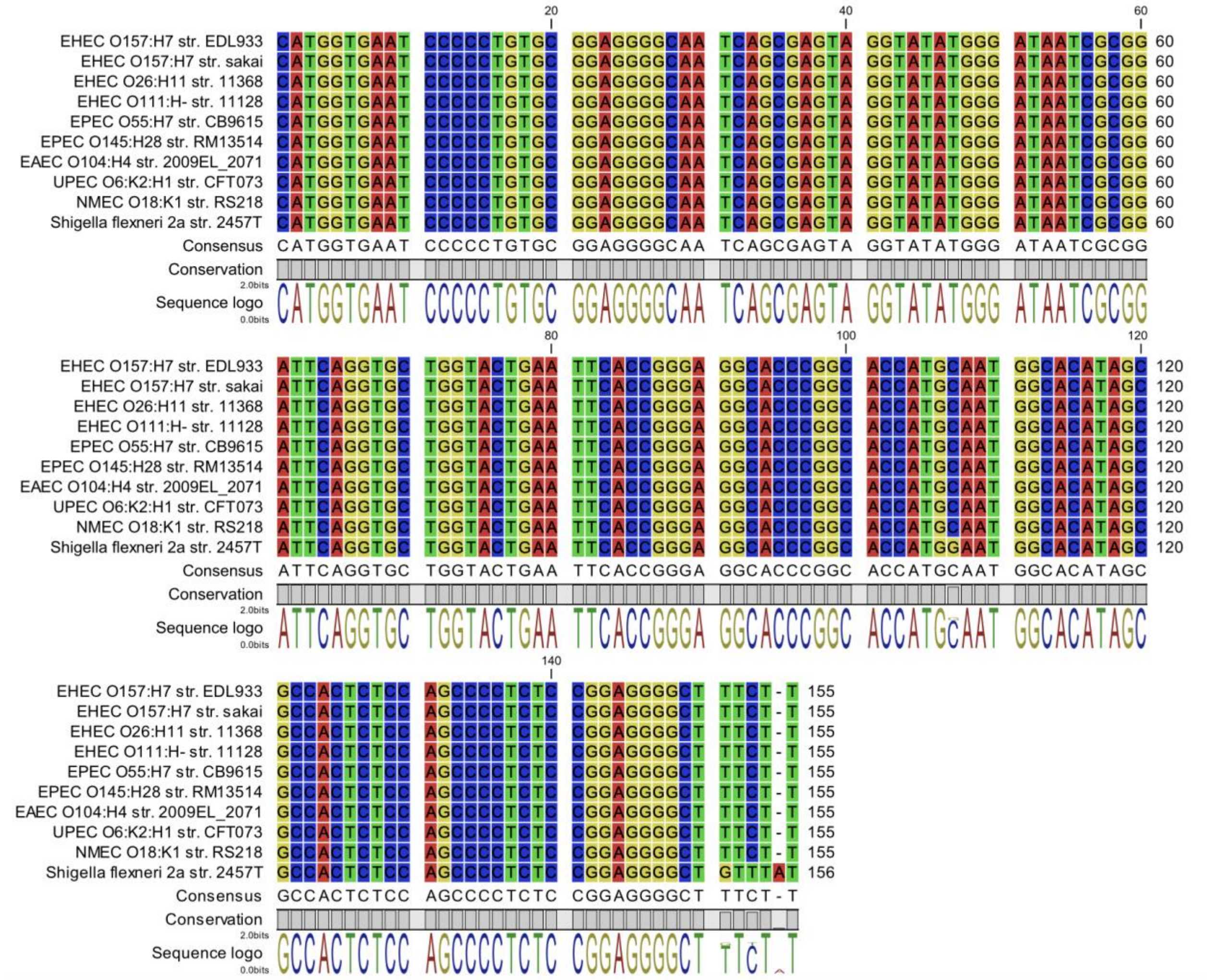

Table S1. Strains used in this study.

**Strains**	**Description**	**Source**
WT	Wild-type EHEC O157:H7 EDL933	ATCC
Δ*evrS*	EvrS deletion mutant in WT	This work
WT^{NalR}	WT with nalidixic acid resistance, Nal^R	This work
Δ*evrS*^{NalR}	Δ*evrS* with nalidixic acid resistance, Nal^R	This work
pEvrS	Δ*evrS* containing pACYC184-EvrS, Cm^R	This work
Δ*ler*	*ler* deletion mutant in WT	Lab stock
Δ*evrS*Δ*ler*	EvrS and *ler* double deletion mutant in WT	This work
WT+pTrc99a-*z2269*	WT overexpressing *z2269*, Amp^R	This work
Δ*ler*+pTrc99a-*z2269*	*ler* deletion mutant overexpressing *z2269*, Amp^R	This work
Δ*z2269*+pTrc99a-*z2269*-3×FLAG	Δ*z2269* carrying pTrc99a-*z2269*-3×FLAG, Amp^R	This work
WT+pBAD-EvrS	WT with pBAD-EvrS plasmid, Cm^R	This work
WT+pBAD-EvrS+ pBAD24-*z2269-lacZ*	WT with pBAD-EvrS plasmid and pBAD24-*z2269-lacZ* plasmid, Amp^R, Cm^R	This work
WT+pBAD-EvrS+ pBAD24-*z2269*-lacZ*	WT with pBAD-EvrS plasmid and pBAD24-*z2269*-lacZ* plasmid, Amp^R, Cm^R	This work
WT+pBAD-EvrS*+ pBAD24-*z2269-lacZ*	WT with pBAD-EvrS* plasmid and pBAD24-*z2269-lacZ* plasmid, Amp^R, Cm^R	This work
WT+pBAD-EvrS*+ pBAD24-*z2269*-lacZ*	WT with pBAD-EvrS* plasmid and pBAD24-*z2269*-lacZ* plasmid, Amp^R, Cm^R	This work
Δ*z2269*	*z2269* deletion mutant in WT	This work

Δ*z2269*Δ*ler*	*z2269* and *ler* double deletion mutant in WT	This work
BL21 (pET28a-*z2269*)	BL21 containing pET28a-*z2269*, Kan^R	This work
Δ*crp*	*crp* deletion mutant in WT	This work
Δ*pdhR*	*pdhR* deletion mutant in WT	This work
Δ*fnr*	*fnr* deletion mutant in WT	This work
Δ*arcA*	*arcA* deletion mutant in WT	This work
WT+P_{rpoD}-*mCherry*	WT strain with *rpoD* promoter fused *mCherry* on the chromosome	This work
WT + pMS402-P_{EvrS}-*gfp*	WT strain with pMS402 carrying *EvrS* promoter fused *gfp*, Cm^R	This work
WT+pMS402-P_{EvrS}	WT strain containing pMS402-P_{EvrS}, Cm^R	This work

Table S2. Plasmids used in this study.

**Plasmids**	**Description**	**Source**
pKD46	Red recombination plasmid, Amp^R	(Datsenko and Wanner, 2000)
pKD3	Containing a chloramphenicol resistance cassette and the flipase recognition sites, Cm^R	(Datsenko and Wanner, 2000)
pKD4	Containing a kanamycin resistance cassette and the flipase recognition sites, Km^R	(Datsenko and Wanner, 2000)
pCP20	Plamid to remove the antibiotic cassette, Cm^R, Amp^R	(Datsenko and Wanner, 2000)
pACYC184	Expression vector, Cm^R	NEB
pEvrS	pACYC184 carrying EvrS and its own promoter, Cm^R	This work
pMS402	*lux*-based promoter reporter plasmid, Km^R	(Fan et al., 2020)
pMS402-P_{EvrS}	pMS402 carrying the EvrS promoter fused *lux* reporter; Km^R	This work
pMS402-P_{EvrS}-*gfp*	pMS402 carrying EvrS promoter fused *gfp*; Km^R	This work
pBAD33	P_{BAD} promoter-based expression vector, Cm^R	(Gruber and Sperandio, 2015)
pBAD-EvrS	pBAD33 carrying EvrS, Cm^R	This work
pBAD24	P_{BAD} promoter-based expression vector, Amp^R	(Gruber and Sperandio, 2015)
pBAD-EvrS*	pBAD33 carrying EvrS* with point-mutation, Cm^R	This work
pBAD24-*z2269-lacZ*	pBAD24 carrying the *z2269-lacZ* fusion, Amp^R	This work

pBAD24-Z2269*-*lacZ*	pBAD24 carrying the *z2269**-*lacZ* gene with point-mutation, Amp^R	This work
pET-28a	T7 expression vector, Km^R	Novagen
pET28a-*z2269*	pET28a carrying *z2269*, Km^R	This work
pTrc99a	High-copy expression vector, Amp^R	Lab stock
pTrc99a-*z2269*	pTrc99a carrying *z2269*, Amp^R	This work
pTrc99a-*z2269*-3×FLAG	pTrc99a carrying *z2269*-3×FLAG, Amp^R	This work

Table S3. Oligonucleotides used in this study.

Name		Sequences
***Primers for construction of mutant strains*[a]**		
EvrS	F	TGAATCCCCCTGTGCGGAGGGGCAATCAGCGAGTAGGTAGTGTAG GCTGGAGCTGCTTC
	R	AGGGGCTGGAGAGTGGCGCTATGTGCCATTGCATGGTGCATGGGA ATTAGCCATGGTCC
z2269	F	AAGGTGAGTGGTTTATGATTATTCACTTTAATACACCAGGTGTAGG CTGGAGCTGCTTC
	R	GTAATTACCTTGATGCCCGGTATTTGCCGGGCATTTACTATGGGAA TTAGCCATGGTCC
crp	F	CTGGCTCTGGAGAAAGCTTATAACAGAGGATAACCGCGCGTGTAG GCTGGAGCTGCTTC
	R	AAATGGCGCGCTACCAGGTAACGCGCCACTCCGACGGGAATGGGA ATTAGCCATGGTCC
pdhR	F	GACCAATTGACTTCGGCAAGTGGCTTAAGACAGGAACTCGTGTAG GCTGGAGCTGCTTC
	R	TTTACAACATCTTCTGGATAATTTTTACCAGAAAAATCAATGGGAA TTAGCCATGGTCC
fnr	F	TTAAAATTGACAAATATCAATTACGGCTTGAGCAGACCTCGTGTAG GCTGGAGCTGCTTC
	R	TAATGATATGACAGAAGGATAGTGAGTTATGCGGAAAAAAATGGG AATTAGCCATGGTCC
arcA	F	GCTAAAAAGCGCCGTTTTTTTTGACGGTGGTAAAGCCGACGTGTAG GCTGGAGCTGCTTC
	R	CTTCCTGTTTCGATTTAGTTGGCAATTTAGGTAGCAAACAATGGGA ATTAGCCATGGTCC

Primers for confirmation of mutant strains		
EvrS	F	ATTTTCCCTGGCTCGCTT
	R	GCAGAGTGATTACTGTCGGAT
z2269	F	AACGGGCGATATTACGAAC
	R	ACTAACCACATCATTGTTCT
crp	F	AGCGGGAAGCATATTTCG
	R	GATAAATCAGTCTGCGCCAC
pdhR	F	GCACAGTTTCATGATTTCAATC
	R	CTCTCGCCGGAAAGCTCA
fnr	F	ATTCAGTCTGGCGGTTGTG
	R	GCCTTGCTCAGTGATGGTATAA
acrA	F	GTCCAGTTCCCAACCATTGA
	R	GGGTAAGAACGGTCTTCTGTTAG
Primers for cloning		
pACYC184-EvrS	F	CGAAGCTTCATGGTGAATCCCCCTGTGC
	R	CCGATATCAAGAAAGCCCCTCCGGAGAG
pBAD-EvrS	F	CGGGACGTCCATGGTGAATCCCCCTGTGC
	R	CCCGAATTCAAGAAAGCCCCTCCGGAGAG
pBAD24-Z2269-*lacZ*	F	CGGGCTAGCAAAAGCTTATTTTCAGCTTTAA
	R	CCCGGATCCTTCGCGTGTTTTCTGTCCAA

pMS402-P_{EvrS}-*lux*	F	GGCTCGAGTTCTAAGCAATCGGTCACTGG
	R	CCGGATCCCCAGTTTCTTTTAACAAACT
pET28a-*z2269*	F	CATGCCATGGcaATGCATTTACGTCATCTGTTTTC
	R	CGGGATCCCAGCGCAATACGAATCACATC
pTrc99a-*z2269*	F	CCCGAATTCATGCATTTACGTCATCTGTTTTC
	R	CGCGGATCCAGCGCAATACGAATCACATC
pTrc99a-*z2269*-3×FLAG	F	CCCGAATTCATGCATTTACGTCATCTGTTTTC
	R	CGCGGATCCTTACTATTTATCGTCGTCATCTTTGTAGTCGATATCATGATCTTTATAATCACCGTCATGGTCTTTGTAGTCACGATTGTATTGCTGGTA
Primers for qRT-PCR analysis		
rrsH	F	GAAAGCGTGGGGAGCAAAC
	R	ACATGCTCCACCGCTTGTG
eae	F	GACGGTAGTTCACTGGACTTCTT
	R	TCGCCACCAATACCTAAACG
tir	F	AAAGCAGCAGGCGAAGAGG
	R	TCGGCACCTGCGAATCAT
ler	F	CAGGAAGCAAAGCGACTG
	R	ACCAGGTCTGCCCTTCTT
escT	F	GCAATAGATGCGGCTGGAC
	R	TCGGCTTGTAATGGTAATATCTCG

escC	F	GACCAAAATGTTGTCGTCCCA
	R	AGGTTACCGCTTCGCTCG
escN	F	AGGTTTTCTTGTTGCCTTTTGA
	R	TCTCCATTGGTCTGCCTATGC
espB	F	AAAACTCCTCGGCAAGATGG
	R	AATAATCCCGCCAACCAAAG
EvrS	F	GGAGGGGCAATCAGCGAG
	R	GGAGAGGGGCTGGAGAGT
z1789	F	GGATATTATTATTTTTAAG
	R	GCCCTTCATTTTTCAGTT
z1793	F	ATCCGCGCATTAAACAGCT
	R	TGGCCGACACAGCCGTACT
z5516	F	GATCTGCCGTTGCAGCCGAT
	R	GAAGGTGGCGTCGTTAACAC
z0776	F	CAACCTGCTCGATATGAGTGG
	R	TTTTACTTACAAAGTTAT
z3901	F	CCAGGTTTTCCAGAAGTTCA
	R	GCGGCCTGAGGTGTTAGAAG
z1614	F	TTAAACTGGACGCCGTCCCGT
	R	GGTTATCGTCATTAACCAGAC

z1175	F	AAAAATGTCCTGGTGGGCC
	R	GAAATCTGAATCGCCGCGCAC
z1307	F	CAGCACGCTGTTTCACGTT
	R	GCGCCGTGCTCAGTCTGTTG
z4241	F	TAAAGATCCAGCCACCTGCA
	R	ACTGCGCGGTTGCCGAAT
z6078	F	TAATTGTTAACTGGTTAACGT
	R	GTTACTGATAGTGACACC
z4541	F	CGGCTTACGAACTTTGACC
	R	GGAACTGGCGCTAGAAATGT
z2077	F	GACACACAATTTCTTAAGTG
	R	CTTTTCCCTGCTGAGAGGG
z5918	F	GCCAATGGTACCGCTATC
	R	TCGTTATGGCAAAAGATTTG
z2269	F	AATGCTTGCGCTGGTGCG
	R	GCGCACGCCTGATTACTA
z2171	F	GGTCCTGGCCAACTAATTGAT
	R	GTATGCTGGATCGCCTGGTC
gfp	F	CAGGAACGCACCATCTTCTT
	R	CACCTTGATGCCGTTCTTCT

mCherry	F	GACTTCTTCAAGTCCGCCAT
	R	TGTGGCTGTTGTAGTTGTACTC
P_{ler}	F	GCTTGGTTTTTATTCTGTTTTATTTGT
	R	ACATCTATTTCATCAAACAACCACC
Primers for RACE analysis		
5'RACE for EvrS	1[a]	TCCGGAGAGGGGCTGGAG
	2[a]	GTGGCGCTATGTGCCATTGC
3'RACE for EvrS	1	TGAATCCCCCTGTGCGGAGGG
	2	AGCACCTGAATCCGCGAT
Probes for Northern blotting		
EvrS	TCAGTACCAGCACCTGAATCCGCGATTATCCCATATACCTACTCGC	
5S rRNA	GAGTCCCCACACYACCAYCGGCGCTACGGCGTTTCACTTC	
Primers for introducing point mutations		
z2269-lacZ*	F	TAAAGTGAATAATCATAAACCA
	R	ATACACCAGGTGTAATCCTTC
EvrS*	F	ATACCTACTCGCTGATTG
	R	TATATGGGATAATCGCGGATT

F, forward; R, reverse; *a*, 1, outer primer; 2, nested primer.

Table S4. Potential EvrS targets predicted by TargetRNA2 (Kery et al., 2014)**.**

**Gene**	**Function**	**Position[a]**	**Energy (kcal/mol)**	***P*-value**
z5502 (*ptsA*)	PEP-protein phosphotransferase system enzyme I	-33 ~ -20	-15.89	0.0001
z5516 (*argC*)[b]	N-acetyl-gamma-glutamyl-phosphate reductase	-10 ~ -20	-13.71	0.002
z2223	hemin-binding lipoprotein	-72 ~ -58	-12.96	0.004
z0776 (*ybeD*)[b]	hypothetical protein	-79 ~ -61	-12.85	0.004
z3901 (*trmD*)[b]	tRNA (guanine-N(1)-)-methyltransferase	-7 ~ +6	-12.75	0.004
z3441 (*bcr*)	bicyclomycin/multidrug efflux system protein	-80 ~ -65	-12.15	0.007
z1614 (*terD_2*)	phage inhibition, colicin resistance and tellurite resistance protein	-80 ~ -62	-11.71	0.009
z1175 (*terD*)	phage inhibition, colicin resistance and tellurite resistance protein	-80 ~ -62	-11.71	0.009
z4290 (*endA*)	DNA-specific endonuclease I	-66 ~ -48	-11.46	0.011
z1307 (*ompA*)[b]	outer membrane protein OmpA	-79 ~ -67	-11	0.014
z4241 (*gcvH*)[b]	glycine cleavage system protein H	+9 ~ +19	-10.86	0.015
z6078[b]	inhibitor of cell division encoded by cryptic prophage CP-933P	+2 ~ +20	-10.55	0.018
z4541 (*rrmJ*)[b]	23S rRNA methyltransferase	-76 ~ -64	-10.28	0.02
z3982	transporter	-68 ~ -53	-9.83	0.025
z0545 (*lon*)	DNA-binding ATP-dependent protease	-46 ~ -32	-9.49	0.03

z3005 (*yecC*)	amino-acid ABC transporter ATP-binding protein	+4 ~ +14	-9.43	0.031
z2077[b]	hypothetical protein	-53 ~ -39	-9.31	0.032
z0127 (*yacH*)	hypothetical protein	-56 ~ -46	-9.27	0.033
z5918 (*fimH*)[b]	minor fimbrial subunit, D-mannose specific adhesin	-40 ~ -20	-9.21	0.034
z2039	regulator of cell division encoded by prophage CP-933O	+2 ~ +20	-9.08	0.036
z2269[b]	DNA-binding transcriptional regulator	-5 ~ +9	-9.03	0.037
z4706 (*slyX*)	hypothetical protein	-37 ~ -26	-8.91	0.039
z2171 (*marR*)[b]	MarR family transcriptional regulator	+6 ~ +20	-8.57	0.045
z4414 (*ttdA*)	tartrate dehydratase subunit alpha	-41 ~ -30	-8.53	0.045
z4265 (*pgk*)	phosphoglycerate kinase	-10 ~ +4	-8.42	0.047

a, binding regions located in the predicted mRNA targets (relative to start codon); *b*, target genes selected for further RT-qPCR analysis.

References:

Datsenko, K. A., and Wanner, B. L. (2000). One-step inactivation of chromosomal genes in Escherichia coli K-12 using PCR products. *Proc Natl Acad Sci U S A* 97, 6640–6645. doi: 10.1073/pnas.120163297

Fan, L., Wang, T., Hua, C., Sun, W., Li, X., Grunwald, L., et al. (2020). A compendium of DNA-binding specificities of transcription factors in Pseudomonas syringae. *Nature Communications 2020 11:1* 11, 1–11. doi: 10.1038/s41467-020-18744-7

Gruber, C. C., and Sperandio, V. (2015). Global analysis of posttranscriptional regulation by GlmY and GlmZ in enterohemorrhagic Escherichia coli O157:H7. *Infect Immun* 83, 1286–1295. doi: 10.1128/IAI.02918-14

Kery, M. B., Feldman, M., Livny, J., and Tjaden, B. (2014). TargetRNA2: identifying targets of small regulatory RNAs in bacteria. *Nucleic Acids Res* 42, W124–W129. doi: 10.1093/NAR/GKU317
